# Adult type granulosa cell tumor of the testis with a heterologous sarcomatous component: case report and review of the literature

**DOI:** 10.1186/1746-1596-9-107

**Published:** 2014-06-03

**Authors:** Thomas EO Schubert, Robert Stoehr, Arndt Hartmann, Sabrina Schöne, Mathias Löbelenz, Gregor Mikuz

**Affiliations:** 1 Institute of Applied Pathology, Speyer, Germany; 2 Department of Pathology, Friedrich Alexander University, Erlangen, Germany; 3 Department of Urology, Hetzelstift, Neustadt/Weinstrasse, Germany; 4 Institute of Pathology, Medical University Innsbruck, Muellerstrasse 44, A-6020 Innsbruck, Austria

## Abstract

Adult testicular granulosa cell tumors are rare sex cord- stromal tumors of which only 45 have been previously reported. As compared with their ovarian counterparts, these tumors may follow a more aggressive course because the proportion of malignant cases is higher. We report here a unique case of a 78-year Caucasian with a left sided adult type granulosa cell tumor with a heterologous sarcomatous tumor component. A heterologous sarcomatous component has occasionally been observed in ovarian tumors but never in testicular granulosa cell tumors. The sarcomatous component showed a higher number of mitotic figures (1/Hpf) and a marked proliferation rate (up to 50% Ki 67 positive cells) compared with the granulosa type tumor component. CD 99 and the progesterone receptor were positive in both tumor components, inhibin and calretinin only in the granulosa cells, and pancytokeratin only in the sarcomatouse one. Key words: testis - ovary - granulosa cells - sarcoma - inhibin Runing title: testicular sarcomatous granulosa tumor.

**Virtual Slides:**

The virtual slide(s) for this article can be found here: http://www.diagnosticpathology.diagnomx.eu/vs/6959043481207016

## Background

Tumors of sex cord/gonadal stroma are exceedingly rare tumors that, in large series, account for 1.6–6% of adult testicular tumors and occur somewhat more frequently in children [1]. Teilum [2] used the term gonadal stroma tumor as a histogenetic and morphologic designation for tumors of both genders which derive from an undifferentiated gonadal mesenchyme. They arise in the testis as well as in the ovary. However, the incidence of the single histological types differs widely between genders. Leydig cell tumor is the most common stromal tumor of the testis, followed by Sertoli cell tumors. Whereas granulosa cell tumors are typical for the ovaries.

The adult type of granulosa cell tumor is extremely rare in the testis – 46 cases have been described to date (Table 1) [3-31]. However, in most of the reports these tumors are only the object of immunohistochemical or cytogenetical investigations; thus, the clinical data are utterly or partially missing [12,15,17,31]. In contrast to the adult type, the juvenile type of granulosa cell tumor is the most frequent testicular tumor in boys younger than six months.

**Table 1:** A synopsis of the morphological and clinical data of the up to now reported cases of the adult type granulosa cell tumor originated in the testis

**Case**	**Age**	**Testis**	**Signs duration**	**Endocrine symptoms**	**Size cm**	**Follow up**	**Source**
1	35	R	15 y	gynecomastia	9	8.5 y NED	Laskowski [3]
2	21	L	incidental	gynecomastia	microscopic	autopsy finding	Cohen and Diamond [4]
3	53	R	1 y	gynecomastia	>10	NS	Massachusetts General Hospital [5]
4	52	R	5 y	none	13	NS	Melicow [6]
5	41	L	8 y	gynecomastia	10,1	5 mo DOD	Mostofi et al. [7]
6	53	R	2 y	gynecomastia	10	17 y NED	Marshall et al. [8]
7	44	R	few months	none	3,5	3 y NED	Talermann, 1985
8	41	R	NS	none	1.8	NS	Gaylis et al. [11]
9	83	L	NS	none	NS	DOC	Düe et al. [12]
10	61	R	2 mo	none	5	2 y NED	Nistal et al. [13]
11	26	L	7 mo	gynecomastia	10	14 y NED	Matoska et al. [14]
12	NS	NS	NS	NS	NS	NS	Sasano et al. [15]
13	42	L	NS	none	NS	AWD at surgery	Monobe, Manabe [16]
14	57	R	10 y	none	2,5	3 y DOC	Jimenez-Quintero et al. [17]
15	55	L	not known	none	1,3	NS	Jimenez-Quintero et al. [17]
16	60	L	many years	none	7	11 y 2 mo DOD	Jimenez-Quintero et al. [17]
17	39	L	2 y	none	4	3 y NED	Jimenez-Quintero et al. [17]
18	16	L	incidental	none	1,8	4 mo NED	Jimenez-Quintero et al. 1993
19	29	R	incidental	none	7,5	14 mo AWD	Jimenez-Quintero et al. [17]
20	76	L	incidental	none	0,7	1 mo NED	Jimenez-Quintero et al. [17]
21	NS	NS	NS	NS	NS	NS	Renshaw et al. [18]
22	NS	NS	NS	NS	NS	NS	Renshaw et al. [18]
23	NS	NS	NS	NS	NS	NS	Renshaw et al. [18]
24	NS	NS	NS	NS	NS	NS	Renshaw et al. [18]
25	NS	NS	NS	NS	NS	NS	Renshaw et al. [18]
26	NS	NS	NS	NS	NS	NS	Renshaw et al. [18]
27	51	L	2 mo	incidental	7	13 mo NED	Morgan,Brame [19]
28	48	R	3 y	none	5	7 mo NED	Al-Bozom et al. [20]
29	54	L	incidental	none	NS	NS	Wang et al. [21]
30	33	NS	incidental	none	1	NS	Guzzo et al. [22]
31	51	L	incidental	none	NS	6 y AWD	Suppiah et al. [23]
32	59	L	2 y	none	15	4 y NED	Hisano et al. [24]
33	32	L	incidental	none	1,98	NS	Arzola et al. [25]
34	77	L	incidental	none	4	NS	Lopez, [26]
35	45	R	months	none	6,5	2 y NED	Ditonno et al. [27]
36	12	L	5 y	none	10	NS	Gupta et al. [28]
37	55	NS	lung metastases	none			Hammerich et al. [29]
38	28	L	incidental	none	2,6	NS	Song et al. [30]
39	21	L	incidental	none	1	2 y NED	Hanson, Ambaye [31]
40	77	R	NS	NS	2,5	NS	Lima et al. [36]
41	22	L	NS	NS	1	NS	Lima et al. [36]
42	40	L	NS	NS	2,1	NS	Lima et al. [36]
43	78	L	incidental	none	13	23 mo NOD	recent case

We observed an adult type granulosa cell tumor with a heterologous sarcomatous tumor component, a morphological feature rarely observed in ovarian granulosa cell tumors [32-34], and only in one testicular Leydig-Sertoli cell tumor to date [35].

## Case report

**Figure 1:**
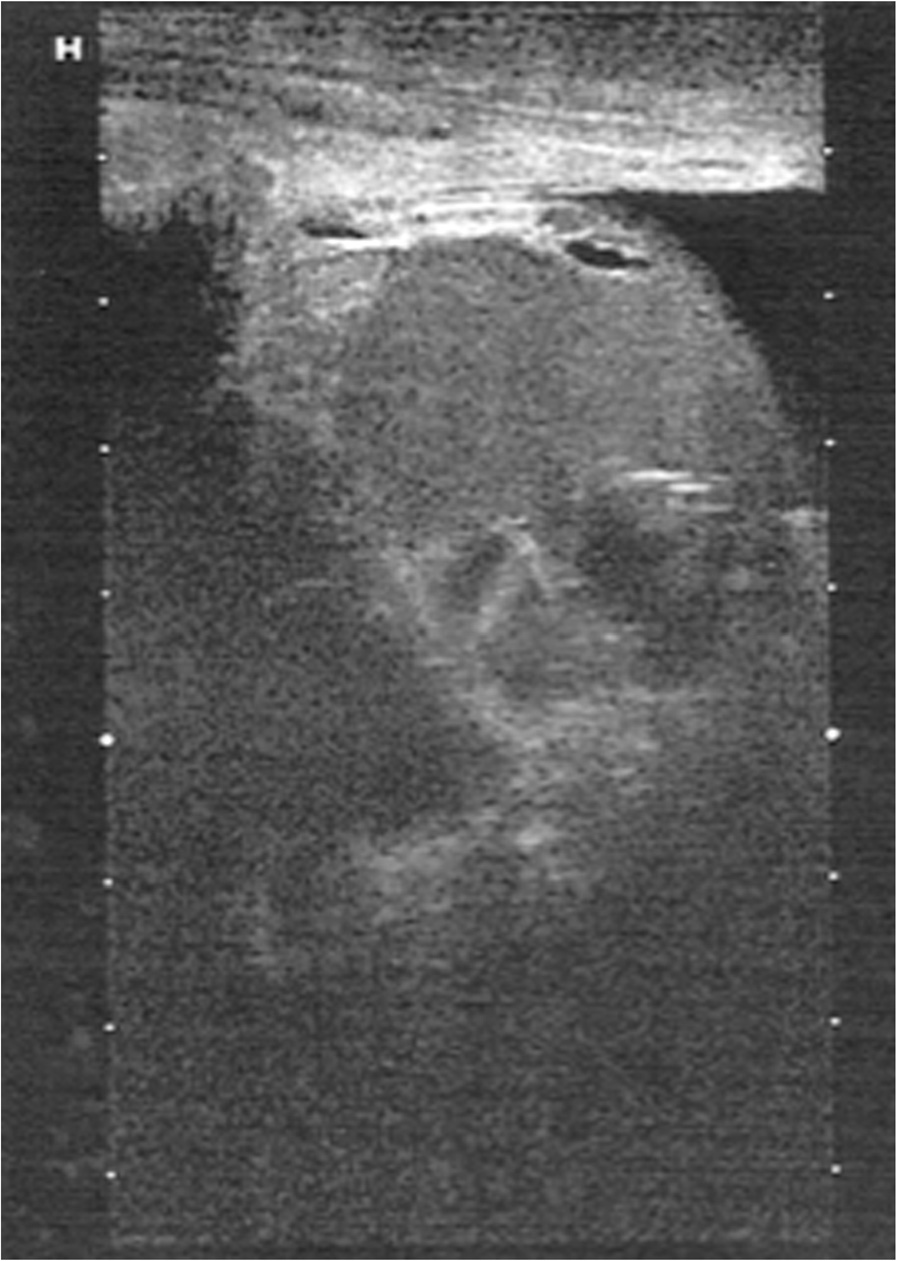
Ultrasound view of a partly cystic tumor.

A 78-year old Caucasian male was admitted to hospital due to acute neurological symptoms. Upon exploration, he reported a history of hypertension and arrhythmia. Upon physical examination, an enlarged left testicle was noted and an inguinal herniotomy scar on the left side. According to statements by the patient, the testicle had started to enlarge a year earlier. After neurological symptoms had remitted, the patient was transferred to the urological unit for further diagnostic procedures. A painless and solid scrotal mass could be palpated on the left side. The left epididymis was not discernible at palpation. The right testicle and epididymis were unremarkable. Upon ultrasonography, the mass had a diameter of 13 cm and contained cystic spaces surrounded by solid tumor structures (Figure 1). There were no enlarged inguinal or abdominal lymph nodes. Upon computed tomography of the chest and abdomen, a cyst measuring 2.5 cm was found in the right kidney, but masses raising suspicion of metastases were not detected. As a consequence of the clinical findings, inguinal semicastration of the left testis was performed. Twenty three months following surgery, the patient is alive and without signs of progressive disease.

**Figure 2:**
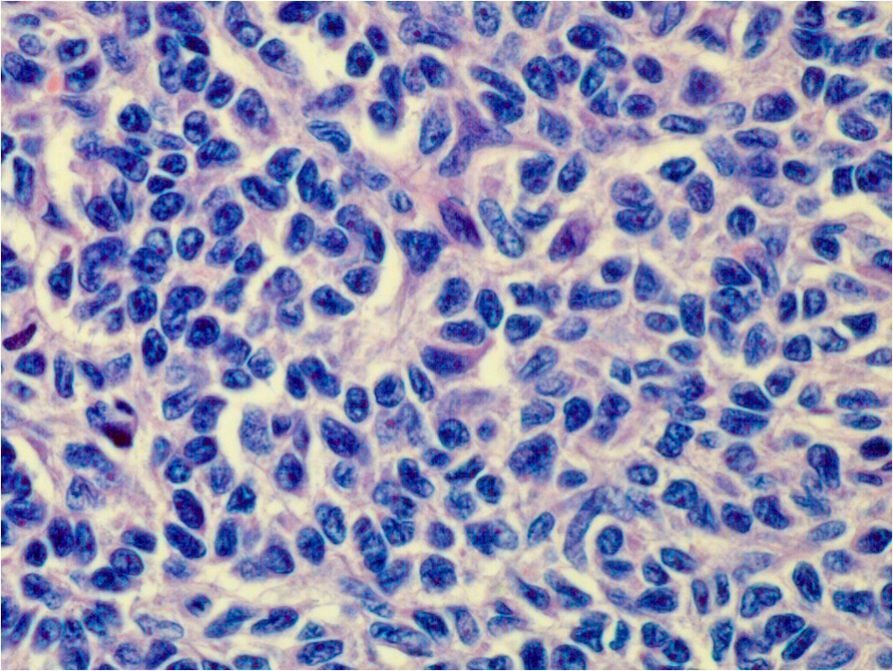
**Tumor forms nest and cord composed of small cells with scant cytoplasm and typical grooved, coffee bean-like nuclei, resembling its ovarian counterpart.** (H&E, orig.magni.x40).

**Figure 3:**
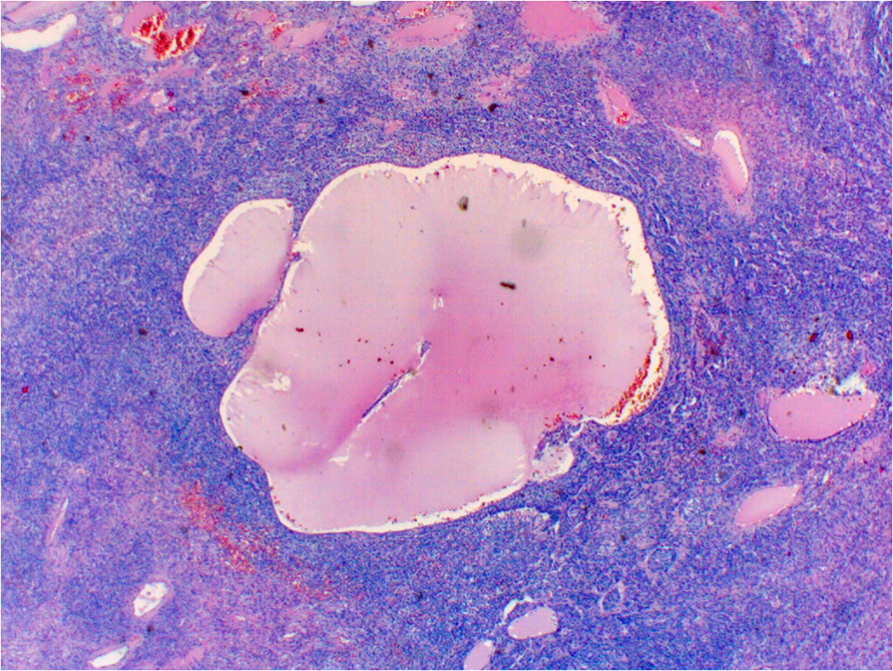
Granulosa cell tumor with cysts resembling the ovarian follicle cysts (H&E, orig.magni.x4).

**Figure 4:**
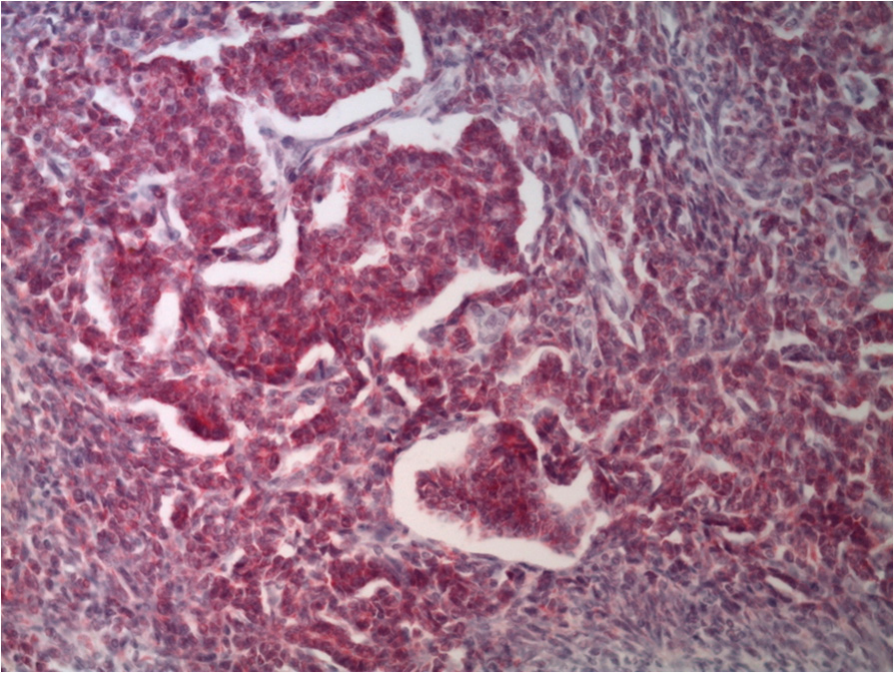
**Higher magnification of the tumor shows cords and follicular nesting of cells similar to those observed in granulosa cell tumor of the ovary.** The tumor cells are inhibin positive (Inhibin immunohistochemistry, orig.magni.x20).

**Figure 5:**
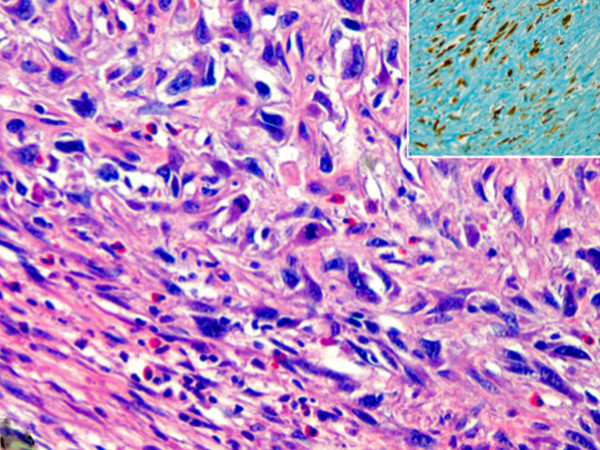
**Sarcomatous area with spindled and stellate cells with atypical mitoses.** The insert shows pancytokeratin (AE1/AE3) positive spindle cells of the sarcomatous area. (H&E and immunohistochemistry,orig.magni.x40.

**Figure 6:**
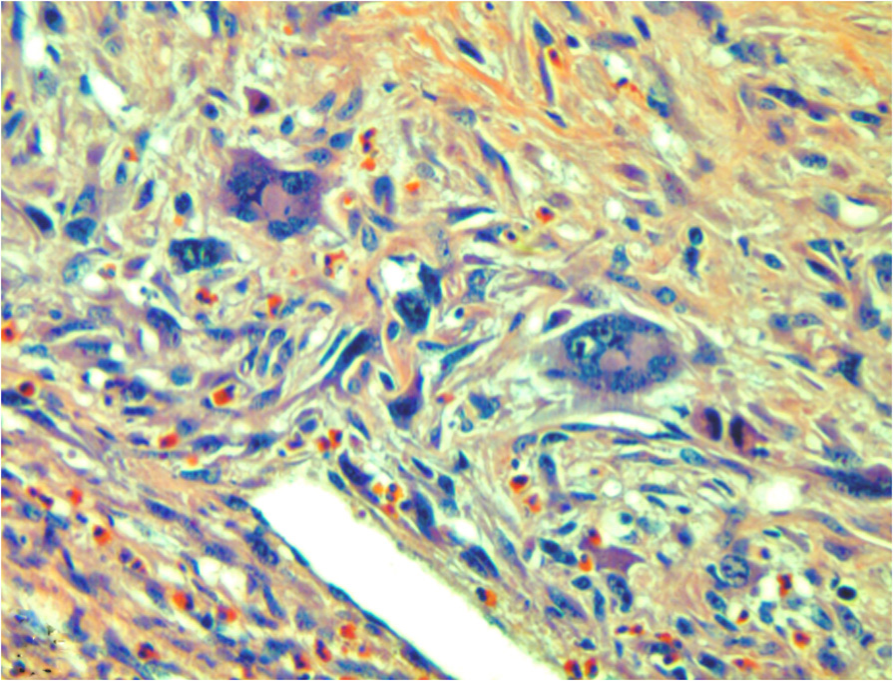
Sarcomatous area with bizarre multinucleated giant cells embedded in a collagenous stroma and a marked infiltration with eosinophils (H&E, orig.magni.x40).

Macroscopically the tumor was composed of solid white or brownish firm masses with multiple cystic spaces.Microscopically the tumor consisted of rather monomorphous typical granulosa cells with scanty cytoplasm and angulated, coffee-bean like, grooved nuclei (Figure 2). The cells grew mainly in solid patterns with some trabecular and gyriform areas and a few cysts lined by tumor cells containing an eosinophilic fluid (Figure 3). Some not well-differentiated microfollicular structures resembling Call-Exner bodies could be found interspersed (Figure 4). Mitotic figures were extremely rare. The tumor showed central necrotic areas. The main part of the tumor was surrounded by fibrotic tissue in which small nests of undifferentiated, spindled cells ordered in sheets were located. These cells did not exhibit any polymorphism and mitotic figures were lacking. At the border of a hyalinized area a proliferation of large, polygonal and spindled cells with many tumor giant cells were present (Figures 5 and 6). The cells had a clear or slightly eosinophylic cytoplasm and markedly polymorphic nuclei with large nucleoli. Almost all mitotic figures (1/hpf) were atypical. Accordingly, the proliferation rate of these sarcomatous cells, assessed by MIB-1 antibodies, was consistently higher (focally > 50%) than that of the typical granulosa cells (<1%). Very large, spindled sarcomatous cells were also found scattered among fibrous collagen rich tissue. The sarcomatous area was infiltrated by a startling number of eosinophilic leucocytes.

**Figure 7:**
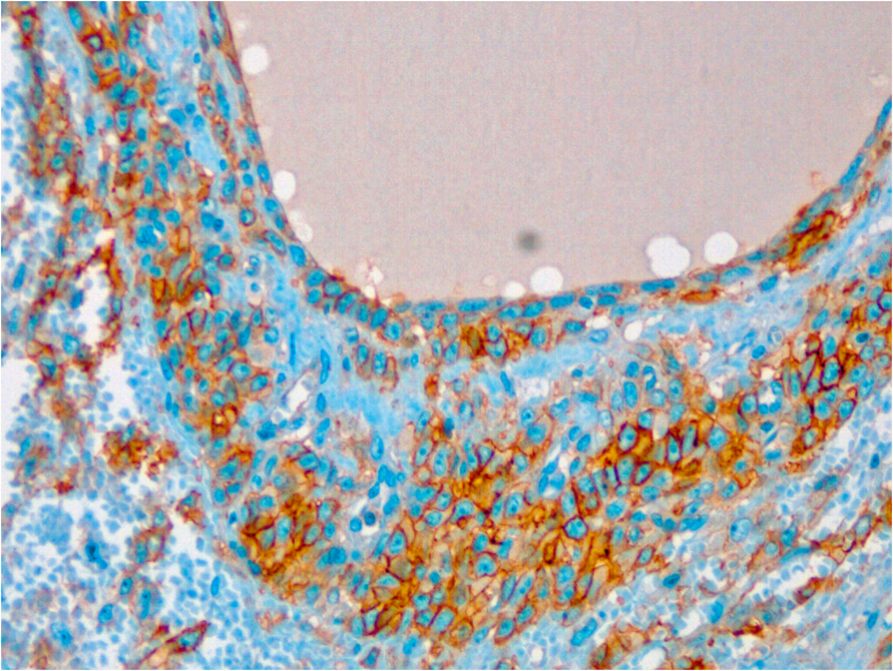
**A follicular structure with surrounding granulosa cells strongly positive for CD 99.** (CD 99 immunohistochemistry, orig.magni.x20) orig.magni.x20).

Immunohistochemically, single typical granulosa cells showed a week reactivity for inhibin and a strong but patchy distributed reactivity for calretinin. Vimentin and CD 99 (Figure 7) as well as progesterone receptor (PR) were strongly expressed in the typical granulosa cells (>70%) as well in the sarcomatous one, whereas androgen- (AR) and estrogen-(ER) receptor antibodies did not show any reactivity. Interestingly, the sarcomatous tumor cells reacted strongly with pancytokeratin antibodies (AE1:AE3) (Figure 5); by way of contrast, the typical granulosa cells were completely unreactive.

Since the FOXL2 402C → G (C134W) mutation is also present in adult-type granulosa cell tumors occurring in men, although in a smaller proportion when compared with the rates reported in women [36], we also performed this mutation analysis. To this end, DNA of the tumor cells was extracted from serial sections (FFPE tissue) after manual microdissection using the Maxwell 16 System (Promega GmbH, Mannheim, Germany) in accordance with manufacturer’s instructions.

Mutation hotspot region containing codon 134 was amplified by PCR (primer sense: 5′- CAACTACTGGACGCTGGACC-3′; primer antisense: 5′-GTTGCCCTTCTCGAACATGTCT-3′). Sequencing analysis was performed using the PyroMark Q24 system (Qiagen, Hilden, Germany) in accordance with manufacturer’s instructions (sequencing primer: 5′- CGCTGGACCCGGCCT-3′). Detailed information about PCR and sequencing conditions is available on request. A FOXL2p.C134W mutation was nonetheless not detectable.

## Discussion

Adult type testicular granulosa cell tumors (GCT) are rare sex cord–stromal tumors. However, subsequent to a thorough study of the literature we were able to find 17 cases above and beyond those referred to in one of the recent literature reviews (28 cases) [31]. Fifteen out of the 45 (33%) reported cases were published after the year 2000, which does not imply that incidence actually increased, but rather, that the tumor is more frequently being recognized.

Morphological diagnosis is based primarily on the typical morphology of the granulosa cells with their coffee-bean like, angulated and grooved nuclei. Macrofollicles ought to be present, the presence of the Call Exner bodies makes correct diagnosis easier; however, they are not always found and thus, are not indispensable to diagnosis [12]. Young and Scully [37] require that “all or almost all” of the tumor should be composed of granulosa cells. In fact, undifferentiated Sertoli cell tumors can have a very similar cytomorphological features but form predominantly ill-defined tubular structures resembling primitive testicular tubules and do not have follicular structures. If the morphological features are ambiguous, the term “incompletely differentiated” (WHO) is preferable. The very rare fibrothecoma of the testis can be also mistaken for undifferentiated sex cord tumor since it manifests the same immunoreactivity as all other stromal tumors. This tumor, however, is composed of highly monotone spindle cells which are embedded in an acellular fibrous stroma [38].

To the best of our knowledge, this is the first case of GCT with heterologous sarcomatous changes observed in the testis. The sarcomatous cells did not show any differentiation, they were negative for desmin and SMA. As in many soft tissue sarcomas, the cells were also positive for pancytokeratin antibodies (AE1:AE3) in our case. With the exception of one case which showed “some evidence of rhabdomyosarcomatous differentiation [32]”, the sarcoma cells in all other cases described in the ovaries did not show any special differentiation [33-35].

One could argue that the observed sarcomatous component originates from a concomitant germ cell tumor growing simultaneously with the granulosa tumor. Quite apart from the fact that the tumor has been entirely processed and no germ cell tumor remnant could be found, a smooth transition from the differentiated to the sarcomatous component could be observed. Moreover, the simultaneous occurrence of germ cell and stromal tumors in the unilateral testis is extremely rare without any common genetic or oncogenetic background [1]. In contrast to the germ cells, the cells of the gonadal stroma tumors are differentiated and not pluripotent; can therefore not give rise to somatic type malignancies (carcinomas, differentiated sarcomas) as the cells of teratomas do. The somatic type malignancies arising in teratomas are well differentiated, completely identical to the tumors originally developing in other organs e.g. soft and nervous tissue, intestine etc. [39], whereas the sarcomas described to date in stromal tumors [32-35] were all composed of undifferentiated spindle cells. It can be assumed that these tumors develop from undifferentiated gonadal stroma.

A molecular characterization of sarcomatous changes in ovarian GCT showed a down-regulation of the GCT-specific genes such as inhibin, estrogen receptor and FSH receptor [35]. A significant up-regulation of genes with an inflammatory response was consistent with the presence “of a marked inflammatory infiltrate”, which was a striking morphological feature in our case as well. However, contrary to these findings of McNeilage et al. [34], the PR receptor was not down-regulated in our case, but immunohistochemically strongly expressed.

Some authors claim that testicular GCT can be mistaken for lymphoma, metastatic carcinoma or even for melanoma [12,35,36], but lymphoma and metastatic carcinoma cells tend to spread in the interstitium between testicular tubules and usually do not form solid tumor masses [1].

Immunohistochemistry is obviously helpful for achieving a correct diagnosis. One must keep in mind, however, that with few exceptions all types of sex cord/gonadal stroma tumor cells show reactivity for the same antibodies. By and large, but not in all cases, the tumor cells of GCT are positive for inhibin, calretinin, vimentin and CD 99 [20,31]. According to Renshaw et al. [18] SMA and S-100 are constantly expressed in all testicular sex cord/gonadal stroma tumors, but other authors cannot confirm these findings [20,30]. CD 99 is consistently expressed, whereas EMA has never been detected in the different types of sex cord/gonadal stroma tumors of the testis; these antibodies are thus useful for the differential diagnosis stromal tumor vs. carcinoma metastasis [20,31,40]. Pancytokeratin and desmin are often but not constantly detected [12,20,31]. Also very useful to the diagnosis of these tumors is the detection of estrogen (ER) and progesterone receptors (PR), and in this connection PR are detected more often than ER (75% vs. 50%) [12]. PlAP immunohistochemistry is obviously the best method to distinguish the stromal from the germ cell tumors of the testes. The PR positivity in the sarcomatous part of the tumor presented here is the only evidence that these cells derive from the granulosa cells and not from the supporting stroma.

A more difficult task is the correct classification of poorly differentiated sex cord/stroma tumors, since the granulosa and Sertoli cells can have a similar appearance and can also express all of the immunohistochemical markers discussed above. Almost all Leydig and granulosa-cell tumors, but only about half of Sertoli-cell tumors, react with inhibin and calretinin antibodies [1]. A valuable marker could be the melan-A/MART-1 (A 103) melanoma antibody, which is positive in all Leydig and in many Seroli-cell tumors, but negative in GCTs [41]. CD 56 is constantly expressed in GCT, but has never been tested in the diagnostics of the testicular GCTs. CD56 is a regulator of growth and differentiation in ovarian folliculogenesis [42].

One of the major problems of histological diagnosis of testicular sex cord/stroma tumors is the prediction of malignancy. Atypical mitotic figures occur more frequently and the mitotic index is significantly higher in malignant tumors; vascular invasion, infiltrative margins, and necrosis are also associated with malignancy, but are not very reliable [1] when used as a single criterion. The only reliable predictor of malignancy in GCT as in all other stromal tumors is a tumor diameter > 5 cm [1,17,31].

Due to the few cases reported, our knowledge of the biological behavior and the clinical features of this tumor is still fragmentary. The average age of the diseased men is 45 years (Range 12 – 83 years) with an accumulation (45%) of cases in the 5^th^ and 6^th^ decade (Table 1). In 65.5% of cases (21/32) the tumors were smaller than 2 cm (1.7 cm in median, range: 0.7 – 15 cm). Endocrine symptoms (gynecomastia, loss of libido and potency) were observed only in six cases, 26 cases did not have such symptoms and in the other cases this clinical information is missing (for references, see Table 1). In the known cases the duration of clinical signs ranged from a few months to 10 years! Nineteen case reports included a follow up: 2 patients died of disease, one of them 11 years after diagnosis [7,17], 3 patients died of unrelated diseases [12,20], 3 patients were alive with disease, one of them 6 years after surgery [16,17,23]. The longest follow-up of one of the 13 patients who were alive without evidence of disease was 14 years [14].

Even this small series of cases shows that testicular adult type GCTs behave in an aggressive manner in one quarter of cases. The clinical problem is that there is no specific therapy for these tumors. Various chemotherapeutic agents and regimens have been used for patients with metastatic disease with limited or no success. The “prophylactic” retroperitoneal lymphadenectomy is an option which is very successful in stage I diseases, but not in the higher stages when the lymph nodes are already affected [43].

In summary, we present a unique case of a testicular granulosa cell tumor of adult type with a sarcomatous component whose impact on the clinical course is unknown.

## Conclusion

The correct classification of poorly differentiated sex cord/stroma tumors can be difficult. Adult type testicular granulosa cell tumors (GCT) are rare sex cord–stromal tumors challenging the diagnostic skills of surgical pathologists. Rarely, these tumors can display a sarcomatous component as described in the reported case.

## Consent

Written informed consent was obtained from the patient for publication of this case report.

## Competing interests

The authors declared no potential conflicts of interest with respect to the research, authorship, and/or publication of this article.

## Authors’ contributions

GM and TS carried out histological and immunohistochemical analysis of the tumor. GM wrote the pathological part of the manuscript and TS helped to draft the manucript. RS and AH performed molecular analysis of tumor tissue and took part in drafting the molecular part of the manuscript. ML and SS carried out clinical diagnostics and wrote the clinical part of the manuscript. All authors read and approved the final manuscript.

## Authors’ information

TS is Head of the Institute of Applied Pathology, Speyer (Germany).

RS is consultant for molecular diagnostic pathology, Department of Pathology, Friedrich Alexander University, Erlangen (Germany).

AH is Head of the Department of Pathology, Friedrich Alexander University, Erlangen (Germany).

SS was consultant urologist of the Department of Urology, Hetzelstift, Neustadt/Weinstrasse (Germany).

ML is Head of the Department of Urology, Hetzlestift, Neustadt (Germany).

GM is Professor Emeritus of Pathology, Medical University Innsbruck (Austria).

## References

[B1] Mikuz G Colecchia M Mikuz G Tumor of sex Cord/Gonadal Stroma 2007 London: Clinical Pathology of Urological Tumors.Informa.healthcare

[B2] Teilum G Special Tumors of the Ovary, and Testis 1976 2 Copenhagen: Munks- gard

[B3] Laskowski J Feminizing tumor of the testis: general review with case report of granulosa cell tumor of the testis Endokrynol Pol 1952 3 337 343

[B4] Cohen J Diamond J Leontiasis ossea, slipped epiphyses and granulosa cell tumor of the testis with renal disease: report of a case with autopsy findings Arch Pathol 1953 56 488 500 13091594

[B5] Case record of the Massachusetts General Hospital (Case no. 41471) N Engl J Med 1955 253 926 931 13272816 10.1056/NEJM195511242532108

[B6] Melicow MM Classification of tumors of the testis: a clinical pathological study based on 105 primary and 13 secondary cases in adults and 3 primary and 4 secondary cases in children J Urol 1955 73 547 574 14368687 10.1016/S0022-5347(17)67439-1

[B7] Mostofi FK Theiss EA Ashley DJB Tumors of the specialized gonadal stroma in human male patients: androblastoma, Sertoli cell tumor, granulosa theca cell tumors of the testis, and gonadal stromal tumor Cancer 1959 12 944 957 14424645 10.1002/1097-0142(195909/10)12:5<944::aid-cncr2820120515>3.0.co;2-t

[B8] Marshall FF Kerr WS Jr Kliman B Scully RE Sex cord-stromal (gonadal stromal) tumors of the testis: a report of 5 cases J Urol 1977 117 180 184 833962 10.1016/s0022-5347(17)58389-5

[B9] Talerman A Pure granulosa cell tumour of the testis. Report of a case and review of the literature Appl Pathol 1985 3 117 122 3842072

[B10] Lawrence WD Young RH Scully RE Talerman A, Roth LM Sex Cord Stromal Tumors 1986 Churchill Livingston: Pathology of the testis and its adnexa

[B11] Gaylis FD August C Yeldandi A Nemcek A Garnett J Granulosa cell tumor of the adult testis: ultrastructural and ultrasonographic characteristics J Urol 1989 141 126 127 2642309 10.1016/s0022-5347(17)40617-3

[B12] Düe W Dieckmann KP Niedobitek G Bornhöft G Loy V Stein H Testicular sex cord stromal tumour with granulosa cell differentiation: detection of steroid hormone receptors as a possible basis for tumour development and therapeutic management J Clin Pathol 1990 43 732 737 2212065 10.1136/jcp.43.9.732 PMC502751

[B13] Nistal M Lazaro R Garcia J Paniagua R Testicular granulosa cell tumors of the adult type Arch Pathol Lab Med 1992 16 284 287 1536615

[B14] Matoska J Ondrus D Talerman A Malignant granulosa cell tumor of the test is associated with gynecomastia and long survival Cancer 1992 69 1769 1772 1551061 10.1002/1097-0142(19920401)69:7<1769::aid-cncr2820690719>3.0.co;2-v

[B15] Sasano H Nakashima N Matsuzaki O Kato H Aizawa S Sasano N Nagura H Testicular sex cord-stromal lesions: immunohistochemical analysis of cytokeratin, vimentin and steroidogenic enzymes Virchows Arch A Pathol Anat Histopathol 1992 421 163 169 1381130 10.1007/BF01607050

[B16] Monobe Y Manabe T Malignant sex-cord stromal tumor of the testis: report of a case with special reference to its unusual intracytoplasmic substructures Jpn J Clin Oncol 1992 22 414 420 1283994

[B17] Jimenez-Quintero LP Ro JY Zavala-Pompa A Amin MB Tetu B Ordoñez NG Ayala AG Granulosa cell tumor of the adult testis: a clinicopathologic study of seven cases and a review of the literature Hum Pathol 1993 24 1120 1225 8406422 10.1016/0046-8177(93)90193-k

[B18] Renshaw AA Gordon M Corless CL Immunohistochemistry of unclassified sex cord-stromal tumors of the testis with a predominance of spindle cells Mod Pathol 1997 10 693 700 9237180

[B19] Morgan DR Brame KG Granulosa cell tumour of the test is displaying immunoreactivity for inhibin BJU Int 1999 83 731 732 10233595 10.1046/j.1464-410x.1999.00069.x

[B20] Al-Bozom IA El-Faqih SR Hassan SH El-Tiraifi AE Talic RF Granulosa cell tumor of the adult type: a case report and review of the literature of a very rare testicular tumor Arch Pathol Lab Med 2000 124 1525 1528 11035589 10.5858/2000-124-1525-GCTOTA

[B21] Wang BY Rabinowitz DS Granato RC Sr Unger PD Gonadal tumor with granulosa cell tumor features in an adult testis Ann Diagn Pathol 2002 6 56 60 11842380 10.1053/adpa.2002.30607

[B22] Guzzo T Gerstein M Mydlo JH Granulosa cell tumor of the contralateral testis in a man with a history of cryptorchism Urol Int 2004 72 85 87 14730174 10.1159/000075281

[B23] Suppiah A Musa MM Morgan DR North AD Adult granulosa cell tumour of the testis and bony metastasis. A report of the first case of granulosa cell tumour of the testicle metastasising to bone Urol Int 2005 75 91 93 16037716 10.1159/000085936

[B24] Hisano M Souza FM Malheiros DM Pompeo AC Lucon AM Granulosa cell tumor of the adult testis: report of a case and review of the literature Clinics (Sao Paulo) 2006 61 77 78 16532229 10.1590/s1807-59322006000100013

[B25] Arzola J Hutton RL Baughman SM Mora RV Adult-type testicular granulosa cell tumor: case report and review of the literature Urology 2006 68 1121.e13-6 17095056 10.1016/j.urology.2006.06.029

[B26] López JI Adult-type granulosa cell tumor of the testis. Report of a case Tumori 2007 93 223 224 17557577 10.1177/030089160709300223

[B27] Ditonno P Lucarelli G Battaglia M Mancini V Palazzo S Trabucco S Bettocchi C Paolo SF Testicular granulosa cell tumor of adult type: a new case and a review of the literature Urol Oncol 2007 25 322 325 17628299 10.1016/j.urolonc.2006.08.019

[B28] Gupta A Mathur SK Reddy CP Arora B Testicular granulosa cell tumor, adult type Indian J Pathol Microbiol 2008 51 405 406 18723972 10.4103/0377-4929.42536

[B29] Hammerich KH Hille S Ayala GE Wheeler TM Engers R Ackermann R Mueller-Mattheis V Malignant advanced granulosa cell tumor of the adult testis: case report and review of the literature Hum Pathol 2008 39 701 709 18304605 10.1016/j.humpath.2007.09.015

[B30] Song Z Vaughn DJ Bing Z Adult type granulosa cell tumor in adult testis: report of a case and review of the literature Rare Tumors 2011 3 e37 22355492 10.4081/rt.2011.e37 PMC3282442

[B31] Hanson JA Ambaye AB Adult testicular granulosa cell tumor: a review of the literature for clinicopathologic predictors of malignancy Arch Pathol Lab Med 2011 135 143 146 21204721 10.5858/2009-0512-RSR.1

[B32] Susil BJ Sumithran E Sarcomatous change in granulosa cell tumor Hum Pathol 1987 18 397 399 3557443 10.1016/s0046-8177(87)80172-7

[B33] Kabukcuoglu F Sungun A Sentürk BA Evren I Ilhan R Mixed germ cell tumor of the ovary with sarcomatous component Pathol Oncol Res 2001 7 60 62 11349223 10.1007/BF03032607

[B34] McNeilage J Alexiadis M Susil BJ Mamers P Jobling T Laslett G Trajstman A Fuller PJ Molecular characterization of sarcomatous change in a granulosa cell tumor Int J Gynecol Cancer 2007 17 398 406 17316362 10.1111/j.1525-1438.2006.00865.x

[B35] Gilcrease MZ Delgado R Albores-Saavedra J Testicular Sertoli cell tumor with a heterologous sarcomatous component: immunohistochemical assessment of Sertoli cell differentiation Arch Pathol Lab Med 1998 122 907 911 9786352

[B36] Lima JF Jin L de Araujo AR Erikson-Johnson MR Oliveira AM Sebo TJ Keeney GL Medeiros F FOXL2 mutations in granulosa cell tumors occurring in males Arch Pathol Lab Med 2012 136 825 828 22742556 10.5858/arpa.2011-0355-OA

[B37] Young RH Scully RE Testicular Tumors 1990 Chicago: ASCP Press

[B38] Sourial MW Sabbagh R Doueik A Ponsot I A 17 year old male with a testicular fibrothecoma: a case report Diagn Pathol 2013 8 152 24044431 10.1186/1746-1596-8-152 PMC4015605

[B39] Mikuz G Colecchia M Teratoma with somatic-type malignant components of the testis. A review and an update Virchows Arch 2012 461 27 32 22622519 10.1007/s00428-012-1251-x

[B40] Gordon MD Corless C Renshaw AA Beckstead J CD99, keratin, and vimentin staining of sex cord-stromal tumors, normal ovary, and testis Mod Pathol 1998 11 769 773 9720506

[B41] Yao DX Soslow RA Hedvat CV Leitao M Baergen RN Melan-A (A103) and inhibin expression in ovarian neoplasms Appl Immunohistochem Mol Morphol 2003 11 244 249 12966351 10.1097/00129039-200309000-00007

[B42] Völker HU Engert S Cramer A Schmidt M Kämmerer U Müller-Hermelink HK Gattenlöhner S Expression of CD56 isoforms in primary and relapsed adult granulosa cell tumors of the ovary Diagn Pathol 2008 3 29 18613980 10.1186/1746-1596-3-29 PMC2474830

[B43] Mosharafa AA Foster RS Bihrle R Koch MO Ulbright TM Einhorn LH Donohue JP Does retroperitoneal lymph node dissection have a curative role for patients with sex cord-stromal testicular tumors? Cancer 2003 98 753 757 12910519 10.1002/cncr.11573

